# Information properties of morphologically complex words modulate brain activity during word reading

**DOI:** 10.1002/hbm.24025

**Published:** 2018-03-09

**Authors:** Tero Hakala, Annika Hultén, Minna Lehtonen, Krista Lagus, Riitta Salmelin

**Affiliations:** ^1^ Department of Neuroscience and Biomedical Engineering Aalto University Helsinki Finland; ^2^ Aalto NeuroImaging, Aalto University Helsinki Finland; ^3^ Department of Psychology Åbo Akademi University Turku Finland; ^4^ MultiLing Center for Multilingualism in Society across the Lifespan, Department of Linguistics and Scandinavian studies University of Oslo Oslo Norway; ^5^ Cognitive Brain Research Unit, Department of Psychology and Logopedics, Faculty of Medicine University of Helsinki Helsinki Finland; ^6^ Department of Political and Economic Studies University of Helsinki Helsinki Finland

**Keywords:** computational linguistics, computational modeling, language, MEG, Morfessor, morphology, N400m, orthography, surprisal

## Abstract

Neuroimaging studies of the reading process point to functionally distinct stages in word recognition. Yet, current understanding of the operations linked to those various stages is mainly descriptive in nature. Approaches developed in the field of computational linguistics may offer a more quantitative approach for understanding brain dynamics. Our aim was to evaluate whether a statistical model of morphology, with well‐defined computational principles, can capture the neural dynamics of reading, using the concept of surprisal from information theory as the common measure. The Morfessor model, created for unsupervised discovery of morphemes, is based on the minimum description length principle and attempts to find optimal units of representation for complex words. In a word recognition task, we correlated brain responses to word surprisal values derived from Morfessor and from other psycholinguistic variables that have been linked with various levels of linguistic abstraction. The magnetoencephalography data analysis focused on spatially, temporally and functionally distinct components of cortical activation observed in reading tasks. The early occipital and occipito‐temporal responses were correlated with parameters relating to visual complexity and orthographic properties, whereas the later bilateral superior temporal activation was correlated with whole‐word based and morphological models. The results show that the word processing costs estimated by the statistical Morfessor model are relevant for brain dynamics of reading during late processing stages.

## 1 INTRODUCTION

The neural processing related to visual word recognition is generally thought to include functionally distinct processing stages, most notably analysis of visual features and letters, access to morphological or lexical units, and activation of their meaning (Coltheart, Rastle, Perry, Langdon, & Ziegler, 2001; Hillis & Rapp, 2004). However, a more detailed account of the computational aspects of these processes is still lacking.

An interesting category of computationally explicit models has been developed within the domain of computational linguistics. Statistical models that are based on unsupervised learning are especially relevant as they are based on mechanisms similar to those of Hebbian learning (Hebb, 1949; Smith, 2011) and therefore have a clear parallel in neural processing. These models could prove useful also for the neuroscience of language if one can establish a justifiable relationship between the models and relevant measures of brain activation. Such a link could arise from the principle of optimization, which has been the basis of many successful applications in the field of natural language processing (NLP; Berger, Pietra, & Pietra, 1996; Hirschberg & Manning, 2015). The principles of optimization and efficiency have also been suggested to guide the neural learning process and organization of brain functions (Friston, 2010). Assuming that efficiency serves as a guiding principle also in the implementation of neural computations involved in reading, NLP models may be able to predict neuroimaging data and provide useful descriptions of the underlying processes. Moreover, describing behavioral and neural data by measures derived from statistical properties can complement description based solely on formal linguistic rules.

In the present study, we used magnetoencephalography (MEG) to quantify the millisecond‐scale neural dynamics of visual word recognition, and relate these results to word measures from a NLP model that optimizes the representation of words. Recognition of morphologically complex words is an important aspect of reading that could be addressed by models emerging from recent efforts in NLP.

A complex word, for example, “builders” consists of multiple morphemes. A morpheme is defined as the smallest meaningful unit of language, which can either stand alone in the form of a monomorphemic word (e.g., “build”) or be bound to the root (e.g., “‐er” and “‐s”). Alongside the NLP model we used more traditional psycholinguistic variables that have been related to visual, orthographic, morphological and lexical processing, thereby linking the present results to previous psycholinguistic literature.

A central question in the recognition of complex words is whether the brain decomposes the word into morphological constituents and then recombines the morphemes into a unified semantic meaning (Fruchter & Marantz, 2015; Taft, 2004; Taft & Forster, 1975), or whether most or all words are represented as whole forms in the mental lexicon (Butterworth, 1983). It seems that at least some mechanism to process words as a compilation of separable parts is required because in highly synthetic languages, such as Finnish, a single root word can have 150 different paradigmatic forms and the total number of possible words is counted in millions (Karlsson, 1983). In these types of languages, storing every possible whole word form in the mental lexicon seems like an uneconomical strategy for the neurocognitive system. However, the decomposition and recombination of morphological constituents may also pose additional processing costs, as suggested by longer reaction times and fixation durations to morphologically complex words than frequency‐matched monomorphemic words (Hyönä, Laine, & Niemi, 1995; Hyönä, Bertram, & Pollatsek, 2005; Lehtonen & Laine, 2003; Soveri, Lehtonen, & Laine, 2007). An optimized model for human word recognition may therefore call for a combination of decomposed and full‐form representations.

NLP algorithms that employ statistical machine learning have shown that morpheme‐like units of representation may emerge from requirements of efficiency in information processing. Morfessor is a data‐driven NLP model that has been successful in inducing morphology from raw text data without a priori linguistic knowledge (Creutz & Lagus, 2007). The model utilizes general learning principles instead of explicit linguistic rules. It is based on the minimum description length (MDL) principle (Rissanen, 1978), and is essentially a packing algorithm that seeks to build an optimally compact and descriptive lexicon of units, called morphs, for describing the training corpus. The Morfessor model thus represents a compromise between views arguing for word representation in full word forms and those that suggest mandatory word decomposition: a particular word can be represented as a full form or decomposed into morphemes depending on which representation optimizes the overall storage and processing efficiency. The morphs discovered by Morfessor can be whole words or sometimes resemble linguistic morphemes, but they are not determined by explicit linguistic rules.

In order to relate predictions of the Morfessor model to brain imaging data, we assume that the brain of an experienced reader has adapted to the statistical regularities of written language, and that the neural activation reflects this adaptation. We can examine this idea by employing tools from the mathematical theory of communication (Shannon, 1948). In information theory, surprisal (also known as self‐information) is defined as an aspect of a probabilistic event that measures the minimum effort needed to communicate the occurrence of that event, and it is quantified by the negative log probability. When the communication is optimized, commonly occurring events require less computational capacity than rare or surprising events that are associated with high information content and high processing requirements. The reading process may be viewed as an optimized communication channel from text to the brain, and the surprisal of a written word is thus related to the minimum processing requirements. This assumption relates to the Bayesian brain hypothesis which proposes that the brain can minimize the free energy, and thus the effort, by representing sensory inputs in an optimal Bayesian fashion, that is, the neural system is organized based on an internal model of the world that is constantly optimized to minimize the long‐term average of surprisal (Friston, 2010; Friston, 2012). Surprisal can be related to the minimum neural activation strength needed to encode the information, as postulated by the efficient coding hypothesis (Barlow, 1961; Linsker, 1990). Given that increases in the MEG signal likely reflect increased neuronal processing, higher surprisal values should be linked with enhanced MEG amplitudes.

The Morfessor model defines a word's surprisal as the sum of the surprisal of its constituent morphs, and can be seen as an estimate of the minimum processing requirement needed in the brain if words are represented as independent morpheme‐like units. The Morfessor values have been shown to correlate with reaction times (RTs) in a lexical decision task better than simple psycholinguistic parameters such as word length or word frequency (Virpioja, Lehtonen, Hultén, Salmelin, & Lagus, 2011; Virpioja et al., 2017). It is, however, still unclear whether the predictive power of Morfessor in RTs is linked to a particular stage of the word recognition process.

In line with the information processing framework, we hypothesize that the salient word‐evoked activations that appear at different time windows and cortical areas correspond to different aspects of information representation and optimization, and the strength of the activation is proportional to the amount of information. For example, the low‐level visual features or orthography can be very similar between two words (e.g., “current” and “currant”) but their corpus‐based word frequencies differ by orders of magnitude (121 vs. 1 per million; Davies, 2010). If these types of qualitatively different information properties are linked to spatio‐temporally distinct brain activations, one can compare the predictive power of models that capture different aspects of stimulus‐related information, and thereby approximate what type of model is most similar to the internal model operating at the neuronal population level.

Earlier MEG studies have identified three temporally and spatially distinct components of brain activation with salient functional roles that are thought to reflect successive stages in the reading process. A midline occipital response peaking at around 100 ms after stimulus onset captures processing related to basic visual features (Tarkiainen, Helenius, Hansen, Cornelissen & Salmelin, 1999; Cornelissen, Tarkiainen, Helenius, & Salmelin, 2003; Wydell, Vuorinen, Helenius, & Salmelin, 2003). It is followed by a letter‐string sensitive activation peaking at around 150 ms in the occipito‐temporal cortex with left‐hemispheric dominance (Tarkiainen et al., 1999; Gwilliams, Lewis, & Marantz, 2016). Activity in the inferior temporal cortex and fusiform area between 150 and 200 ms has also been reported to be sensitive to orthographic and morphological properties of letter strings, supporting the so‐called morpho‐orthographic segmentation hypothesis in which decomposition is based on visual word forms and takes place prior to lexical access (Solomyak & Marantz, 2010; Zweig & Pylkkänen, 2009). After 250 ms, the left superior temporal cortex shows a sustained response that usually reaches the maximum at around 400 ms (often referred to as N400m). This activation has been linked to lexical, semantic, phonological and morphosyntactic analysis in word processing (Halgren et al., 2002; Helenius, Salmelin, & Connolly, 1998; Salmelin, 2007; Service, Helenius, Maury, & Salmelin, 2007). Several studies have also found the earliest evidence of morphological processing in this cortical area (Cavalli et al., 2016; Fruchter & Marantz, 2015; Vartiainen et al., 2009; Whiting, Shtyrov, & Marslen‐Wilson, 2015).

In the present study, we extract these well‐established patterns of brain activation during a lexical decision task and compare them to the Morfessor estimate as well as to psycholinguistic variables that have been linked to visual word processing (Hauk, Davis, Ford, Pulvermüller, & Marslen‐Wilson, 2006; Pylkkänen & Marantz, 2003; Wydell et al., 2003). These variables seek to estimate aspects of low level visual (image complexity, word length), orthographic (bigram frequency), morphological (Morfessor, lemma frequency, lemma transition probability) or lexical (surface frequency) processing. To assess how Morfessor estimates and/or each of the psycholinguistic variables are related to the brain activity, we employ item‐level correlation and multiple regression analysis. As each item is presented only once to avoid confounding effects of item repetition in the lexical decision task, the signal‐to‐noise ratio of the item‐level responses is enhanced by averaging the single trials per each word across the participants.

The interpretation of the results on words is aided by a comparison to the results of a similar analysis on the pseudowords that were presented in the lexical decision task. Non‐lexical variables, such as those related to visual features and orthography should be comparable for real words and pseudowords. However, neural processing related to any form of meaning should dissociate between real words and pseudowords.

We predict that the early stages of neural activity during reading will correlate best with surprisal values in the visual or orthographic measures, whereas the later activation is better captured by morphological and lexical variables, with higher neural activation associated with higher surprisal values. Of specific interest is whether the letter‐string response will be better explained by orthographic or morphological variables and to what degree the sustained left temporal response will capture both morphological and lexical information measures. Moreover, any unique predictive power of the Morfessor measure would suggest that a particular brain response is linked to processing of morpheme‐like representations and that it is possible, at least to some extent, to find such units by requiring compactness of representation using the minimum description length principle.

## 2 METHODS

### 2.1 Participants

A total of 23 Finnish‐speaking participants were recruited for the experiment. Three participants were excluded due to a low number (less than 290/360) of artifact‐free trials with a correct response. Data from 20 participants were thus included in the analysis: 11 female, age 20–37 (mean 24.4, *SD* = 6.4), all right‐handed as assessed by the Edinburgh Handedness Inventory (Oldfield, 1971), with no reported neurological problems. The participants gave their informed consent, and were reimbursed for their time. The study was approved by the ethics committee of the Hospital District of Helsinki and Uusimaa.

### 2.2 Experimental design

**Figure 1:**
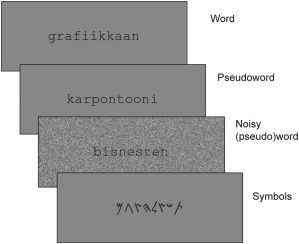
Experimental stimuli. Examples of the four functionally distinct stimulus categories: words, pseudowords, symbols strings, and (pseudo)words embedded in Gaussian random noise. Each trial consisted of a fixation cross that appeared for 500 ms, followed by a single stimulus that was displayed for 1,500 ms

The experimental setup was a visual lexical decision task. The stimuli were 1,440 unique items from four categories: words, pseudowords, symbol strings as well as words and pseudowords masked with Gaussian noise (for examples, see Figure 1). The pseudowords, symbol strings and noisy stimuli were used for the functional localization step of the study. The word set consisted of 360 Finnish nouns taken from the Morpho Challenge 2007 corpus consisting of 55 million word tokens (2.2 million unique), which is part of the Wortschatz collection (Quasthoff, Richter, & Biemann, 2006). The set included monomorphemic, as well as inflected and derived multimorphemic words. It also had a high variance across several psycholinguistic variables to allow correlational analyses. The word length varied from 4 to 16 letters (mean 10.3, *SD* 2.8), frequency of word occurrence was 0.018–127 per million (mean 1.87, *SD* 10), and the number of linguistic morphemes was 1–5 (mean 2.8, *SD* 1.1; note that the root word also counts as a morpheme). The pseudowords consisted of 360 letter strings generated randomly using a probabilistic n‐gram model trained on a Finnish text corpus. The pseudowords followed the phonotactic rules of the Finnish language, that is, they were pronounceable and resembled real words but carried no meaning. The length distribution of the pseudoword set matched that of the word set.

The noise‐embedded items consisted of 60 real words and 60 pseudowords that were masked by a rectangular patch of Gaussian random noise. The level of noise was such that the word was just barely readable. The symbols were 120 letter strings in the Phoenician alphabet, with a length distribution matching that of the word set. The characters had visual qualities akin to letters, but were not easily confused with Finnish alphabets in typical fonts. None of the participants reported familiarity with ancient Phoenician writing systems. Moreover, 120 random filler words from the corpus were added to counterbalance for the 120 symbol strings in order to equalize the number of word and non‐word items in the experiment.

The stimuli were projected on a screen placed at a distance of 140 cm from the participant's eyes. The items were presented in black font (lower case Courier New monospaced) on a gray background. The visual angle per letter was 0.41°. Each trial consisted of a centered fixation cross, displayed for 500 ms, followed by a stimulus item displayed for 1,500 ms. The participant's task was to identify whether the item was a real Finnish word or not, as fast and accurately as possible. Responses were given via an optical response device that reacted to index finger lift. The “yes”/“no” responses were randomly assigned to the left/right index finger, balanced across the participants. The responses did not affect the course of the experiment, nor was feedback provided during the task. If the correct response was not given within the 1,500 ms period when the word was displayed, or if the response was given accidentally before 350 ms (median RT minus three times the median absolute deviation), the trial was rejected. Each item was shown only once to each participant. The RTs from the correct responses were collected and used for a behavioral assessment.

The stimulus order was randomized and the presentation divided into six blocks, lasting around 7 min each, with short resting breaks between the blocks. The order of the blocks was balanced across the participants using the Latin square design.

### 2.3 Measurements

Cortical activity during task performance was recorded with a Vectorview whole‐head MEG system (Elekta Ltd., Helsinki, Finland) at the MEG Core, Aalto NeuroImaging. The system employs a total of 306 sensors at 102 locations, with each location equipped with two planar gradiometers in an orthogonal configuration and one magnetometer. The MEG data was band‐pass filtered at 0.03–200 Hz and sampled at 1,000 Hz.

Four electrodes were attached next to the eyes to record vertical and horizontal electro‐oculograms (EOG) for detection of blinks and eye movements. Head position was measured with the help of indicator coils placed on the scalp and their locations determined with respect to predefined fiducial points to allow MEG co‐registration with participant's anatomical magnetic resonance images (MRIs).

The anatomical MRIs, with 1 × 1 × 1 mm^3^ resolution, were obtained on a separate occasion using the T1 MPRAGE sequence on the Siemens Skyra 3T MRI scanner of the Advanced Magnetic Imaging Centre, Aalto NeuroImaging.

### 2.4 MEG data analysis

As each stimulus word was shown only once during the experiment to avoid priming effects, and the signal‐to‐noise ratio thus could not be enhanced by averaging within each participant, the responses were averaged across different participants, instead. For this reason, we first located functionally, temporally and spatially corresponding responses of written word processing (at about 100, 150, 400 ms) in individual participants. Each of these responses were then averaged over participants, separately for each word.

The continuous raw data was cleaned from external interference with the spatiotemporal Signal Space Separation method (Taulu & Simola, 2006) and low‐pass filtered to 40 Hz. Epochs were extracted using a time window from −200 to 800 ms with respect to the stimulus onset. Trials contaminated by blink or muscle artifacts were excluded. Rejection criteria were >150 μV for the EOG electrodes and >3,000 fT/cm for the MEG gradiometers. Only trials where the participant responded correctly were included in the further analysis.

The sensor‐level summation of electromagnetic fields was disentangled into its underlying source‐level components using the equivalent current dipole model (ECD; Hämäläinen, Hari, Ilmoniemi, Knuutila, & Lounasmaa, 1993; Salmelin, 2010). The aim was to isolate at least the four well‐established components consistently identified in visual word recognition: the occipital response at around 100 ms, the left occipito‐temporal response at 150 ms, as well as the sustained left temporal response reaching the maximum at around 400 ms after stimulus onset, together with its right‐hemisphere counterpart.

The ECD models were first constructed separately for each participant based on the averaged field pattern of all word items, following the standard procedure (Salmelin, 2010). The field patterns were calculated using the data from planar gradiometers that are more sensitive to the cortical currents near the sensors and less sensitive to distant noise sources than the magnetometers.

Each source‐level model consisted of 4–9 ECDs that adequately reproduced the observed whole‐head field patterns with goodness‐of‐fit >80%. The four ECDs of interest were identified based on their location in the individual anatomical MRI, peak time, and functional behavior with respect to the different stimulus categories using the criteria presented in (Tarkiainen et al., 1999). The occipital activity peaked at around 100 ms (for individual peak latencies, range 90–131 ms, mean 104 ms, SD 11 ms). This ECD was found in 17/20 participants, and was stronger for noisy than noiseless stimuli. An ECD in the left‐hemispheric occipitotemporal area reached the maximum at around 150 ms (range 140–189 ms, mean 154 ms, SD 12 ms) and exhibited a stronger response to letter than symbols strings (identified in 15/20 participants). A left temporal component with sustained activation peaking around 400 ms (range 314–478 ms, mean 389 ms, SD 52 ms) was found in 19/20 participants. A functionally similar component was also found in the right hemisphere at around 400 ms (range 331–519 ms, mean 411 ms, SD 63 ms) in the same participants. These temporal cortex sources differentiated words from pseudowords and their activation was diminished for non‐word symbol strings.

**Figure 2:**
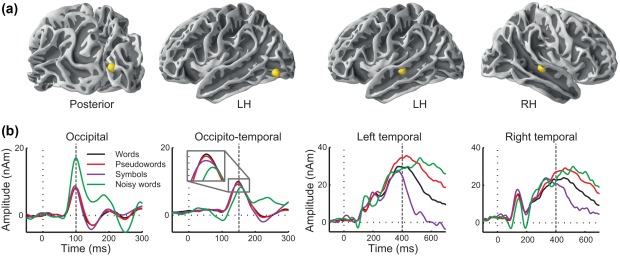
Source modeling. (a) The final source model for each participant consisted of four temporally, spatially and functionally identified ECDs. The locations of ECDs were identical across subjects, but the orientation was determined individually. (b) The ECD amplitude time courses averaged for each stimulus category and across participants highlight the distinct functional roles of the different ECDs. The dashed vertical line represents the time at which the ECD was localized

Subsequently, in order to maximize the comparability of the source model across the participants, an averaged multi‐ECD model was constructed: The coordinates of all identified occipital, occipito‐temporal, and bi‐lateral temporal components in each participant were projected from individual MRIs to the Freesurfer “fsaverage” average brain template (Dale, Fischl, & Sereno, 1999; Dale et al., 2000; Fischl, Liu, & Dale, 2001). The ECD locations of each component in the resulting 4‐ECD model were averaged across the participants in the common coordinate system and then morphed back into individual coordinates of each participant (Figure 2a). Finally, the ECD orientations were optimized individually for a best match to the field pattern at preselected time points: for the occipital response at 100 ms, for the occipito‐temporal response at 150 ms, and for both temporal cortex responses at 400 ms. The cortical dynamics of each participant was thus described by a 4‐ECD model even if all the corresponding ECD components were not included in the original individual ECD model of a given participant.

To verify that this process retained the response functionality of the original individual ECD models that included the corresponding components, the source activation strengths for each stimulus category were averaged over participants (Figure 2b). In agreement with the representative individual models, the averaged occipital amplitude peaked at 100 ms and was strongly increased for noisy stimuli. The occipito‐temporal peak at 150 ms was strongest for text items and attenuated for symbols. This attenuation, depicted in the Figure 2b inset, is slight but significant (*t* test *p* < .001). The effect is somewhat smaller than previously reported (Tarkiainen et al., 1999), which may be due to our choice of using Phoenician alphabets as symbols. They are more similar to letters than the geometric shapes used in previous studies.

For the bilateral temporal sources, the activation diminished rapidly for symbols after 350 ms and was stronger and longer‐lasting for pseudowords than words.

For single‐item correlation analysis, the source amplitudes for the individual words were estimated, per participant, by averaging activation in pre‐defined time windows based on earlier literature: for occipital response at 80–120 ms, occipito‐temporal letter‐string sensitive response at 140–200 ms (Salmelin, 2007; Tarkiainen et al, 1999), and for the sustained temporal responses at 300–700 ms (Embick, Hackl, Schaeffer, Kelepir, & Marantz, 2001; Lau, Almeida, Hines, & Poeppel, 2009; Pylkkänen & Marantz, 2003; Vartiainen et al., 2009).

The amplitude values for each response type in the corresponding time‐window were normalized within each participant by taking the z‐score. For each word, the z‐scores were then averaged across the participants. The z‐scores were used instead of absolute values of source amplitudes in order to attenuate inter‐individual variation of a more general nature. These single‐word level measures of brain activation were brought into the linear regression model and evaluated against the values from language models.

### 2.5 Psycholinguistic and NLP variables

In correlating language models with brain activation and RTs, we employ the Shannon's surprisal measure (Shannon, 1948) that defines the surprisal (*I*), associated with an event ω as a $I\left(\omega \right)=-\log\left(P\left(\omega \right)\right),$ where $P\left(\omega \right)$ is the probability of the event. It is expressed as units of information, e.g., bits. The surprisal measure can be obtained from any language model that quantifies the probability of a word in some way. A model that resembles the brain's internal model should correlate better with neural activity than a model that does not. We consider several ways to quantify the word information content with variables that should be sensitive to different levels of abstraction: low level visual features (word length, image complexity), orthographic features (bigram frequency), morphology (the NLP model Morfessor, lemma frequency, lemma transition probability [TPL]), and lexical processing (surface frequency).

Word length corresponds to a simple model that assumes individual letters as basic units of representation and considers all letters equally probable: if the probability of an individual letter is *P*(letter), the surprisal related to a word is $I\left(word\right)=-\log\left({P\left(letter\right)}^{N}\right)\propto N$, that is, proportional to the length (*N*) of the word. As the word length is roughly proportional to the surface‐level visual complexity, the word length model could describe the very early visual processing stages better than models optimized for more abstract features of language. To examine this hypothesis a bit further, we sought to find an alternative way to measure low‐level image complexity. We adopted a practical approach in line with information theoretic framework that makes use of compression algorithms: the gif‐index is defined as the ratio of image file size in compressed (gif format) to size in uncompressed (bitmap) image format. This measure has been found to correlate well with other complexity measures and also with subjective assessments of complexity (Donderi, 2006; Palumbo, Ogden, Makin, & Bertamini, 2014).

We quantify orthographical properties using the mean open bigram frequency which has been proposed to describe part of the processing along the ventral occipito‐temporal cortex (Dehaene, Cohen, Sigman, & Vinckier, 2005; Grainger & Whitney, 2004). The open bigrams capture the relative position of letters in a string by describing a word via ordered letter pairs. For example, the word “take” is represented by TA, TK, TE, AK, AE, and KE. Again, we take negative logarithm to obtain a measure of information.

Our main morphological variable is the Morfessor baseline model (Creutz & Lagus, 2007) that has proven successful in inducing simple morphology from raw text data, independent of the language. The model has, for example, been shown to work well with highly inflected languages such as Finnish and Turkish (Creutz, Lagus, Lindén, & Virpioja, 2005; Mermer & Saraclar, 2011). The model learns the optimal units of representation (“morphs”) from a text corpus. An encoded representation consists of two parts: a model M consists of a lexicon of letter strings, and a message X consists of pointers that refer to the lexicon. In this representation, a word like “build + er” can be expressed by pointers to morphs “build” and “‐er”. These units have their individual surprisal values that are related to their frequency of occurrence in the training corpus. The surprisal of the whole word is the sum of surprisal values of its constituent morphs. As the morph dictionary is quite extensive, it is also possible to express pseudowords using the same model. The pseudowords in our study are generated using n‐gram models that reflect statistical properties of real Finnish words and, consequently, they contain letter snippets that coincide with Morfessor morphs. The pseudowords thus have surprisal values that reflect the degree to which they have common elements with real words.

In order to relate the Morfessor model to other measures of morphology, we also examine lemma frequency which is the total frequency of words sharing a given root. The word lemma should be activated following successful morphological parsing. In addition, we examine TPL, which is defined as surface frequency divided by lemma frequency, that is, it expresses the conditional probability of encountering the whole word form, given the stem (Solomyak & Marantz, 2010).

Finally, surface frequency corresponds to the idea of a mental lexicon that stores whole word forms. This measure can be used as an index of full‐form lexical access. The whole word's surprisal is given by the negative logarithm of surface frequency. The inter‐correlations between these variables are shown in Table 1.

**Table 1:** Correlations between predictor variables

	Image complexity	Word length	‐log bigram frequency	TPL	Morfessor	‐log Lemma frequency	‐log Surface frequency
**Image complexity**	1	0.81**	−0.07	−0.06	0.42**	0.05	0.27**
**Length**		1	0.09	−0.05	0.54**	0.06	0.34**
**‐log bigram frequency**			1	0.06	0.06	−0.08	0.09
**TPL**				1	0.01	0.61**	−0.10*
**Morfessor**					1	0.38**	0.74**
**‐log lemma frequency**						1	0.40**
**‐log surface frequency**							1

*p < .05, **p < .001.

The correlation of the different models to brain activity and RTs (the mean value to each word across individuals) was performed using linear regression. For an initial overview, correlation coefficients of individual predictors to each cortical component were computed with simple linear regression. Next, multiple linear regressions were employed to assess the contribution of the different predictors in conjunction, that is, the source amplitudes and RTs were predicted using a linear combination of variables. Significance of a given variable in the multiple regression model indicates that the variable has unique predictive power that is not explained by its correlation with the other variables.

In order to visualize the brain‐level results, grand‐average waveforms were computed with respect to the predictor variable with highest correlation for a given cortical source. The stimulus words were divided into three bins corresponding to the highest third, lowest third and average values of the predictor, and the grand average responses were plotted over subjects and word bins for each source component.

## 3 RESULTS

All 20 participants included in the analysis performed the task at an acceptable level (at least 80% correct). Mean accuracy was 92% (*SD* 4%), indicating that the participants complied well with the task instructions. The average RT for words was 852 ms (*SD* = 95 ms) and for pseudowords 950 ms (*SD* = 94 ms). Reaction times were significantly correlated with each of the tested variables (*F*(1,358), *p* < .001), with the exception of the TPL, in a simple correlation analysis (Table 2). Morfessor reached the highest correlation (*r* = .61) with RTs. For pseudowords, the RTs were correlated with image complexity, word length and Morfessor values (*F*(1,358), *p* < .001), with word length attaining the highest correlation (*r* = .64).

**Table 2:** Correlation coefficients r of word measures to source component amplitudes and reaction times

Predictor variables	Source component				Reaction times
	Occipital	Occipito‐temporal	Left temporal	Right temporal	
**Words**					
Image complexity	0.21**	−0.11*	0.01	0.12*	0.42**
Length	0.31**	−0.13*	0.07	0.25**	0.57**
‐log bigram frequency	0.003	−0.11*	−0.11*	−0.09	−0.20**
TPL	0.02	−0.01	0.02	0.04	0.06
Morfessor	0.21**	−0.10*	0.32**	0.29**	0.61**
‐log lemma frequency	0.01	−0.03	0.21**	0.17**	0.32**
‐log surface frequency	0.11*	−0.08	0.35**	0.21**	0.55**
**Pseudowords**					
Image complexity	0.28**	−0.14*	0.12*	0.28**	0.50**
Length	0.39**	−0.16*	0.15*	0.33**	0.64**
‐log bigram frequency	−0.1	−0.04	0.05	−0.004	0.04
Morfessor	0.28**	−0.14*	0.17*	0.30**	0.34**

*p < .05, **p < .001.

The multiple regression model (Table 3) was constructed by stepwise regression, that is, adding predictors to the model one by one and estimating the significance of the improvement. This procedure showed that all measures except image complexity and TPL had unique predictive power for word RTs; the complete model (*F*(6,354) = 77, *p* < .001) explained *R* ^2^ = .52 of total variance. The correlation coefficients for predictors, as well as β‐coefficients in multiple regression, attained positive values for word length, Morfessor, ‐log surface and ‐log lemma frequencies, indicating that higher values of information as measured by these variables resulted in longer reaction times. In contrast, the orthographical ‐log bigram frequency showed a negative correlation. For pseudoword RTs, the multiple regression yielded significant results for word length and Morfessor values.

**Table 3:** Multiple regression β coefficients of predictors to source component amplitudes and reaction times. *R* ^2^ is the total variance explained by the complete model

Predictor variables	Source component				Reaction times
	Occipital	Occipito‐temporal	Left temporal	Right temporal	
**Words**					
Image complexity			−0.15^*^	−0.24^*^	
Length	0.30**	−0.15^*^		0.33**	0.36**
‐log bigram frequency		−0.14^*^			−0.11**
TPL					
Morfessor			0.22^*^	0.21**	0.20^*^
‐log lemma frequency					0.14**
‐log surface frequency			0.22^*^		0.21**
Total *R* ^2^	0.09**	0.04^*^	0.15**	0.12**	0.52**
**Pseudowords**					
Image complexity					
Length	0.39**	−0.16^*^		0.34**	0.89**
‐log bigram frequency					
Morfessor			0.17^*^		−0.34**
Total *R* ^2^	0.16**	0.03^*^	0.03**	0.11**	0.46**

*p < .05, **p < .001.

**Figure 3:**
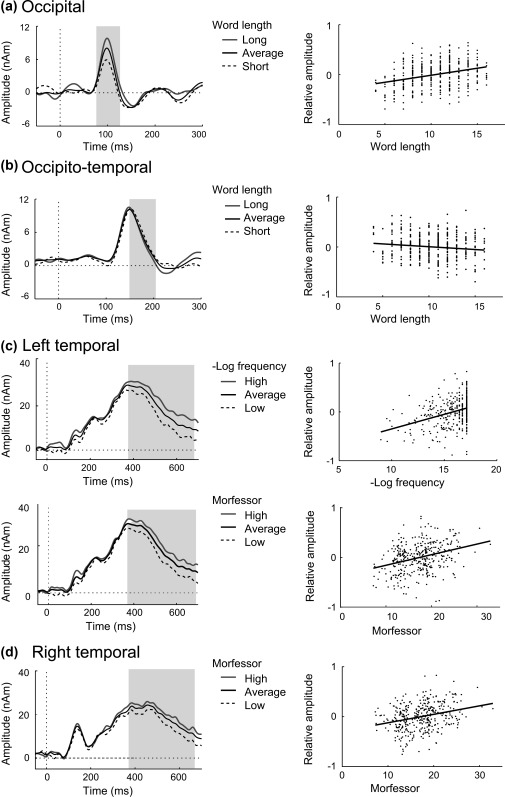
Visualization of how item‐level cortical activations are related to linguistic models, with the highest correlations displayed for each studied response type. The time course of activation is averaged in bins of 60 words (lowest, average, highest values of the model). The scatter plots depict the relative source amplitudes (averaged over the time window marked with gray in the time course) for individual words with respect to the linguistic model

The brain‐level results for each of the four evoked response components of interest (occipital, occipito‐temporal and left and right temporal) are illustrated in Table 2, Table 3 and Figure 3. The amplitude of the occipital response at 80–120 ms after word onset was best correlated with word length (*r* = .31, *p* < .001). Similar results were obtained for pseudowords (Table 2). The source amplitude increased nearly linearly with string length, by approximately 0.5 nAm/letter; single‐item correlations are presented in Figure 3a. Image complexity and Morfessor values also correlated with the occipital response, but became redundant in the multiple regression model.

The amplitude of the occipito‐temporal response at 140–200 ms for words was significantly (*p* < .05) correlated with image complexity (*r* = –.11), word length (*r* = –.13), ‐log bigram (*r* = –.13) and Morfessor (*r* = –.11) by simple correlation analysis (Table 2, Figure 3b). In the multiple regression model, word length and ‐log bigram frequency remained significant (Table 3). For pseudowords, the correlations were significant with respect to image complexity (*r* = –.14), word length (*r* = −0.16) and Morfessor (*r* = .14). The bigram frequency did not reach significance. In multiple regression only length remained significant.

The left‐hemispheric temporal response strength at 300–700 ms was best correlated with the whole‐word frequency (*r* = .35; Table 2), with the cortical activation strength increasing with an increasing value of surprisal (Figure 3c). There was also a significant correlation with lemma frequency (*r* = .21), Morfessor measure (*r* = .32; Figure 3d) and bigram frequency (*r* = –.11). Multiple regression analysis indicated that Morfessor, word frequency and image complexity had unique predictive power; the regression model (*F*(4,354) = 20, *p* < .001), explained *R* ^2^ = .15 of the source variance (Table 3). For pseudowords, the response was significantly correlated with image complexity (*r* = .12), word length (*r* = .15) and Morfessor (*r* = .17). In multiple regression, only Morfessor remained significant.

The right‐hemispheric temporal response strength at 300–700 ms was significantly correlated with Morfessor (*r* = .29; Figure 3e), word length (*r* = .25), word ‐log frequency (*r* = .21) and image complexity (*r* = .12; Table 2). In multiple regression, image complexity, word length, and Morfessor all remained significant, but word frequency became redundant (Table 3). For pseudowords, image complexity (*r* = .28) string length (*r* = .33) and Morfessor (*r* = .30) were significant, but only word length remained significant in multiple regression.

The visualization based on bins of the lowest, middle and highest values for each predictor showed that the amplitude of the occipital response increased with increasing string length (Figure 3a). For the occipito‐temporal source, the effect was visible in the descending slope after the peak (Figure 3b), with increasing letter‐string length associated with a steeper descent, resulting in a negative correlation coefficient in the overall time window.

## 4 DISCUSSION

We investigated the utility of an NLP model for morphological segmentation in predicting cortical activation patterns and RTs during visual word recognition. Models derived from an NLP framework based on optimization principles were hypothesized to mirror efficiency in neural processing. The Morfessor model induces morphology from raw text data instead of relying on predetermined linguistically defined morphs. The surprisal values derived from Morfessor reflect an optimized morph‐based representation of a word and may, as such, correlate with the brain responses related to morphological processing. In comparison, the variables related to image complexity, word length, and bigram frequency should be related to processing of lower‐level visual and orthographic processing. Surface frequency, when the lower‐level effects are factored out, should index activation related to lexical or semantic processing.

We hypothesized that the early occipital activation could be best predicted by word length and image complexity that approximates the surprisal relating to overall visual processing, whereas the later occipitotemporal and superior temporal activations would likely be better accounted for by models using higher levels of abstraction, reflecting processes related to language processing per se.

The behavioral RT results showed that word length, bigram frequency, Morfessor, lemma frequency and surface frequency each provided unique predictive power to reaction time in a multiple regression model. This indicates that each of these variables captured an aspect of the overall reading and decision‐making process that was not fully accounted for by the other variables. When only one predictor was used at a time, Morfessor outperformed the other measures, replicating the results of Virpioja et al. (2011). The good performance of the Morfessor model is especially noteworthy as surface frequency and word length have consistently proven to be some of the best overall predictors of RTs (Keuleers, Lacey, Rastle, & Brysbaert, 2012). We also performed a similar analysis for pseudowords and found RT to be sensitive to length and Morfessor measures in the multiple regression model. The increasing pseudoword length resulted in longer RT; however, increasing Morfessor value, when used in conjunction with string length, was associated with shorter RT. The Morfessor model represents pseudowords using the same optimized units that are used to represent real words. This means that pseudowords that attain lower Morfessor values contain morphemes or text strings that are common in real words. It is likely that the longer reaction times for these pseudowords reflect greater difficulties to reject a pseudoword that has more in common with real words. This conclusion is consistent with the result that pseudowords with a higher number of lexical neighbors elicit longer RTs (Carreiras, Perea, & Grainger, 1997; Holcomb, Grainger, & O'Rourke, 2002). The Morfessor measure could, therefore, be considered a measure of “word‐likeness” when applied to pseudowords.

At the level of the brain, the response amplitude of the 80–120 ms occipital activation increased with increasing number of letters, in line with earlier studies (Assadollahi & Pulvermüller, 2003; Wydell et al., 2003). This amplitude was also correlated with image complexity measured by the gif‐index, as well as surface frequency and the Morfessor measure. All of these four measures give high surprisal values for long words. However, only one of these predictors was significant in the multiple regression model, suggesting that a single underlying factor is responsible for the correlation. The lowest‐level common denominator here is the overall image complexity. This interpretation is further supported by the fact that the response is highly sensitive to addition of noise in the stimuli. Higher visual complexity is related to longer word length, which in turn is correlated with the other variables.

The subsequent 140–200 ms activation in the left inferior occipito‐temporal cortex was identified by its differentiation of alphabetic strings from graphical symbol strings. The letter‐string effect points to a neural population trained by repeated exposure to written text and acting as a bridge or filter between visual and more abstract language processing (Tarkiainen et al., 1999). Part of the present research question was therefore to test to what degree this response is sensitive to orthographic and morphological properties. We found that the response strength was best predicted by word length which was interchangeable with effects of image complexity and Morfessor measures, similarly to the earlier occipital activation. However, these correlations were rather weak. In addition, the letter bigram frequencies provided unique predictive power in the multiple regression model, suggesting that the response is indeed related to abstract orthographic properties rather than low‐level visual features alone. The effect seemed to stem from the descending slope following the peak of the evoked response. A similar result has previously been observed when contrasting consonant strings and (pseudo)words (Whiting et al., 2015). In that study, significant effects emerged between 155 and 230 ms, following the peak response centered at 150 ms.

In the present study, the amplitude of the occipito‐temporal response was negatively correlated with both word length and bigram frequency (the longer the word or lower the mean bigram frequency, the lower the amplitude), which seems to contradict our hypothesis that higher information content leads to more neural activation. These results might be related to activation of specialized “bigram cells”, postulated by Dehaene et al. (2005), located in the left occipito‐temporal sulcus. The bigram cells are thought to be active when the stimulus contains common letter bigrams for which these neurons are tuned. Hence, low bigram frequency results in low activation. Indeed, the response was also found to be reduced when adjacent letters were vertically shifted with respect to each other, which breaks the bigram form (Cornelissen et al., 2003).

We found no independent effects related to word frequency measures, TPL, or Morfessor model in the occipito‐temporal response. Previous studies, on English words, have found support for automatic form‐based decomposition (Fruchter, Stockall, & Marantz, 2013), morphological decomposition indexed by TPL (Solomyak & Marantz, 2010), as well as effect of morphological complexity in a corresponding right hemispheric response (Zweig & Pylkkänen, 2009). However, several other studies have not been able to detect effects of morphology in the letter‐string response. Neither Vartiainen et al., (2009) using Finnish words or Whiting et al., (2015) using a similar contrast with English words found differences in the occipito‐temporal region between simple and complex words. Likewise, a study of morphological priming effects using French words by Cavalli et al. (2016) did not reveal any significant effects of morphology in the posterior temporal cortex. Instead, all three aforementioned studies reported morphological effects after 250 ms in the middle temporal cortex. Thus, the functional role of the occipito‐temporal activity in morphological processing seems to remain somewhat unclear. Interestingly, a recent study, using English words as stimuli and applying a special variant of distributed source modeling, suggests that the occipito‐temporal response may in fact consist of two functionally and temporally distinct components: the first would be associated with orthographic processing and the second with more abstract lexical processing (Gwilliams et al., 2016). In the present study, to ensure robust across‐participants matching, the letter‐string response was modeled as a single source in the more traditional fashion (Tarkiainen et al., 1999), and it may thus not capture a possible second component in a more anterior region that might be linked to lexicality or morphology.

In the bilateral superior temporal cortices, the activation reached its maximum at around 400 ms after stimulus onset. Both hemispheres differentiated between all stimulus types at 300–700 ms, and the response was characterized as a N400m type response (Salmelin, 2007). In the left temporal cortex, all frequency measures and the Morfessor model were positively correlated with the activation strength: activation increased with increasing surprisal values. The type of morphological processing that is described in the Morfessor model thus seems to be reflected in this cortical response. Temporal activation in this time‐window has previously been linked to a wide variety of linguistic and nonlinguistic manipulations (Kutas & Federmeier, 2011; Salmelin, Kujala, & Liljeström, in press), later‐stage word recognition processes (Halgren et al., 2002), and access to semantic‐syntactic representations of morphemes or their recombination to a meaningful whole (Fruchter & Marantz, 2015; Vartiainen et al., 2009). In addition, surprisal, when derived from a sentence context, has been shown to be a good predictor of the N400 amplitude (Frank, Otten, Galli, & Vigliocco, 2015). The present study shows that surprisal is a relevant measure also for predicting the response to isolated words without a surrounding sentence context.

We observed that the overall prediction accuracy for the left temporal responses was improved when using both surface frequency and the Morfessor measure together. This may imply that access to full word forms occurs in parallel with processing of the word's morphological constituents, or that some subset of words is better described by one model. Indeed, many of our low‐frequency words occur only once in the entire corpus and are, therefore, poorly differentiated based on surface frequency but have a smooth distribution on the Morfessor value scale.

In addition, we found that word length and image complexity were good predictors of the left temporal response in the case of pseudowords but performed worse for real words. This suggests that the processing of pseudowords may be primarily linked to letter‐by‐letter or phonological representations which are roughly indexed by word length in the orthographically transparent Finnish language, whereas the representation of real words is related to more abstract linguistic or semantic properties. The Morfessor model also proved to be a good predictor of the temporal responses in case of pseudowords, but the multiple regression model did not determine whether this result was truly independent of the word length effect.

In the right hemispheric N400m type response, reaching its maximum at around 400 ms, the Morfessor model, together with word length, image complexity and the frequency measures were positively correlated with the response amplitude. However, in the multiple regression analysis whole‐word frequency became redundant. This result suggests that the right hemisphere is also actively involved in the morphological processing. Although the right hemisphere has received somewhat less attention in language studies, there have been documented cases where lesions to right hemisphere have resulted in specific inability to produce derivational morphology (Marangolo et al., 2003). Effect of inflectional morphology has also been observed on right‐sided EEG responses (Leinonen et al., 2009). More generally, the right hemisphere has been proposed to become involved when processing requires additional effort (Kircher, Brammer, Tous Andreu, Williams, & McGuire, 2001; Monetta, Ouellet‐Plamondon, & Joanette, 2006; Van Ettinger‐Veenstra, Ragnehed, McAllister, Lundberg, & Engström, 2012) or when semantic complexity increases (Tremblay, Monetta, and Joanette, 2009). In line with this view, the right‐hemisphere activation in the present study could be related to the morphological complexity of words which pose particular demands on semantic integration of the constituents.

Multiple regression analysis of single‐item MEG responses enabled assessment of several predicting variables simultaneously, without prior assumptions that are needed in a univariate approach. Related analysis approaches have been successfully employed before (e.g., Hauk et al., 2006; Solomyak & Marantz, 2009). In the present study, our solution for improving the signal‐to‐noise ratio to the single items was to average single‐item responses across the participants. In order to achieve reliable responses by this approach, the variation between individuals both in terms of amplitude strength and spatial locations need to be accounted for. Here we normalized the amplitude strength for each individual before averaging and sought to equivocate the spatial location by means of functional localizers. It is also worth noting that the amount of explained variance in the MEG responses (*R* ^2^ ∼ .1) was substantially lower than in RTs (*R* ^2^ ∼ .5), despite the fact that MEG presumably captures the subprocesses involved in reading more directly. While MEG provides a more detailed description of when and where different aspects of text are processed in the brain, its signal‐to‐noise ratio is lower than that of the RTs. The reading process in the brain may also entail aspects that are not captured by the evoked responses (i.e., signals phase‐locked to the stimulus timing) but which are included in the all‐encompassing RT measure.

To conclude, the present study offers a methodological example of how a modern NLP model may be used to address questions of language processing in the human brain. The Morfessor model seems to account for brain activation during reading: the observed good predictive power of Morfessor in lexical decision RT (Virpioja et al., 2017; Virpioja et al., 2011) seems to be related to late‐stage morphological processing that is reflected in bilateral temporal cortices from about 300 ms onwards. This suggests that the type of computational properties that are expressed in Morfessor, that is, morpheme‐like units derived via optimization, are also reflected in neural processing. Neural processing thus likely follows some form of optimization, in line with the information‐theory based principle of minimization of effort. Our findings support the view that the brain of an experienced reader has adapted to the statistical regularities of written language, and that the neural activation reflects this adaptation. In the future, Morfessor or similar models could be used, for example, to model how the statistics of native‐language vocabulary can influence the learning and representation of word forms in a new language.
